# MGr1-Antigen/37 kDa laminin receptor precursor promotes cellular prion protein induced multi-drug-resistance of gastric cancer

**DOI:** 10.18632/oncotarget.17795

**Published:** 2017-05-11

**Authors:** Guanhong Luo, Weijie Wang, Qiong Wu, Yuanyuan Lu, Tao Su, Nan Gu, Kai Li, Jingbo Wang, Rui Du, Xiaodi Zhao, Xiaohua Li, Rui Fan, Hongbo Zhang, Yongzhan Nie, Xinmin Zhou, Yongquan Shi, Jie Liang, Xin Wang, Daiming Fan

**Affiliations:** ^1^ State Key Laboratory of Cancer Biology and Xijing Hospital of Digestive Diseases, The Fourth Military Medical University, Xi'an, China; ^2^ Department of Cardiology, Xijing Hospital, The Fourth Military Medical University, Xi'an, China; ^3^ Department of Anesthesiology, Xijing Hospital, The Fourth Military Medical University, Xi'an, China; ^4^ Department of Radiotherapy Oncology, Navy General Hospital, Beijing, China

**Keywords:** PrP^C^, MGr1-Ag/37LRP, gastric cancer, multi-drug-resistance (MDR), apoptosis

## Abstract

Cellular prion protein (PrP^C^), the infective agent of transmissible spongiform encephalopathies, is thought to be related to several cellular physiological and physiopathological processes. We have previously reported that PrP^C^ participates in multi-drug-resistance of gastric cancer. As the salient ligand molecule of PrP for participating in internalization and propagation of the scrapie form of prion protein (PrP^Sc^), 37 kDa laminin receptor precursor protein (37LRP) shared the same gene coding sequence of MGr1-Ag, another protein previously found to be involved in multi-drug-resistance of gastric cancer in our lab. In the present study, we explored whether MGr1-Ag/37LRP contributed to PrP^C^ mediated multi-drug-resistance in gastric cancer. Immunohistochemical staining showed similar expression patterns of MGr1-Ag/37LRP and PrP^C^ in gastric cancer tissue serial sections. Western blot and immunohistochemistry also demonstrated correlative expression of MGr1-Ag/37LRP and PrP^C^ in gastric cancer cell lines. Interaction between MGr1-Ag/37LRP and PrP^C^ in gastric cancer cell lines and gastric cancer tissues were verified by immunofluorescence and co-immunoprecipitation. Furthermore, knockdown of MGr1-Ag/37LRP significantly attenuated PrP^C^ induced multi-drug-resistance by sensitizing drug-induced apoptosis through inhibition of AKT activation. In conclusion, MGr1-Ag/37LRP may interact with PrP^C^ and promote the PrP^C^ induced multi-drug-resistance in gastric cancer through PI3K/AKT pathway. The current study elucidates the mechanism of how PrP^C^ triggers intracellular signaling cascade resulting in multi-drug-resistance phenotype and provides a novel candidate molecular target against gastric cancer.

## INTRODUCTION

PrP^C^ is the cellular form of prion protein, which is generally considered to be the infective agent of transmissible spongiform encephalopathies both in human and animals [1]. As a glycosyl-phosphatidylinositol-anchored membrane bound glycoprotein, PrP^C^ is ubiquitously expressed in mammalian cells especially in neurons and is highly conserved among species [2]. However, the physiological role of PrP^C^ still remains elusive. To date, the putative roles of PrP^C^ are thought to be related to a lot of physiological and physiopathologic processes including cell adhesion, cell growth and proliferation, cell death, signal transduction, as well as cholesterol, iron, zinc and copper metabolism [3–7]. Our previously studies showed that PrP^C^ is highly expressed in gastric cancer tissues and gastric cancer cell lines and has notable effects on tumorigenesis and metastasis of gastric cancer [8, 9]. Moreover, we also previously reported that PrP^C^ is related to multi-drug-resistance with aberrant high expression in Adriamycin(ADR)-resistant gastric adenocarcinoma cell line SGC7901/ADR and Vincristine(VCR)-resistant cell line SGC7901/VCR, [10] which also displayed cross-resistance to other anticancer drugs [11]. Previous data demonstrated that ectopic over-expression of PrP^C^ might induce gastric cancer cells to display significantly enhanced resis tance to chemical therapeutic drugs [12].

To gain a better insight into prion's putative role, studies looking for molecules interacting with prion protein have identified divalent metal ions, several proteins, and nucleic acids [13] Among them, the 37KDa laminin receptor precursor (37LRP) was thought to be a salient ligand of PrP, [14] which plays an important role in internalization, propagation of the PrP^Sc^ and scrapie desease infection [15–17]. 37LRP shares the same gene coding sequence with MGr1-antigen (MGr1-Ag), a novel protein we have found contributes to multi-drug-resistance of gastric cancer [18]. Hypoxia-mediated up-regulation of MGr1-Ag/37LRP occurs via a HIF1-dependent mechanism and contributes to drug resistance in gastric cancers [19].

Since we found that PrP^C^ could promote drug resistance in gastric cancer, questions about how PrP^C^ triggers the intracellular signaling cascade remain. For example, whether there is a binding partner that functions synergistically with PrP^C^ and contributes to the multi-drug-resistance phenotype is unknown. We found that PrP^C^ and MGr1-Ag/37LRP are involved in hypoxia-mediated gastric cancer MDR. Moreover, they were verified to alter the tumorous drug resistant phenotypes through similar pathways [12, 20]. Thus, we presume that MGr1-Ag/37LRP probably is important in the mechanism of PrP^C^ related multi-drug-resistance in gastric cancer. In the present study, we explored whether MGr1-Ag/37LRP contributes to PrP^C^mediated multi-drug-resistance, which may provide an understanding of the specific mechanism of PrP^C^-mediated multi-drug-resistance.

## RESULTS

### MGr1-Ag/37LRP and PrP^C^ are correlatively expressed in gastric cancer tissues and gastric cancer cell lines

**Figure 1:**
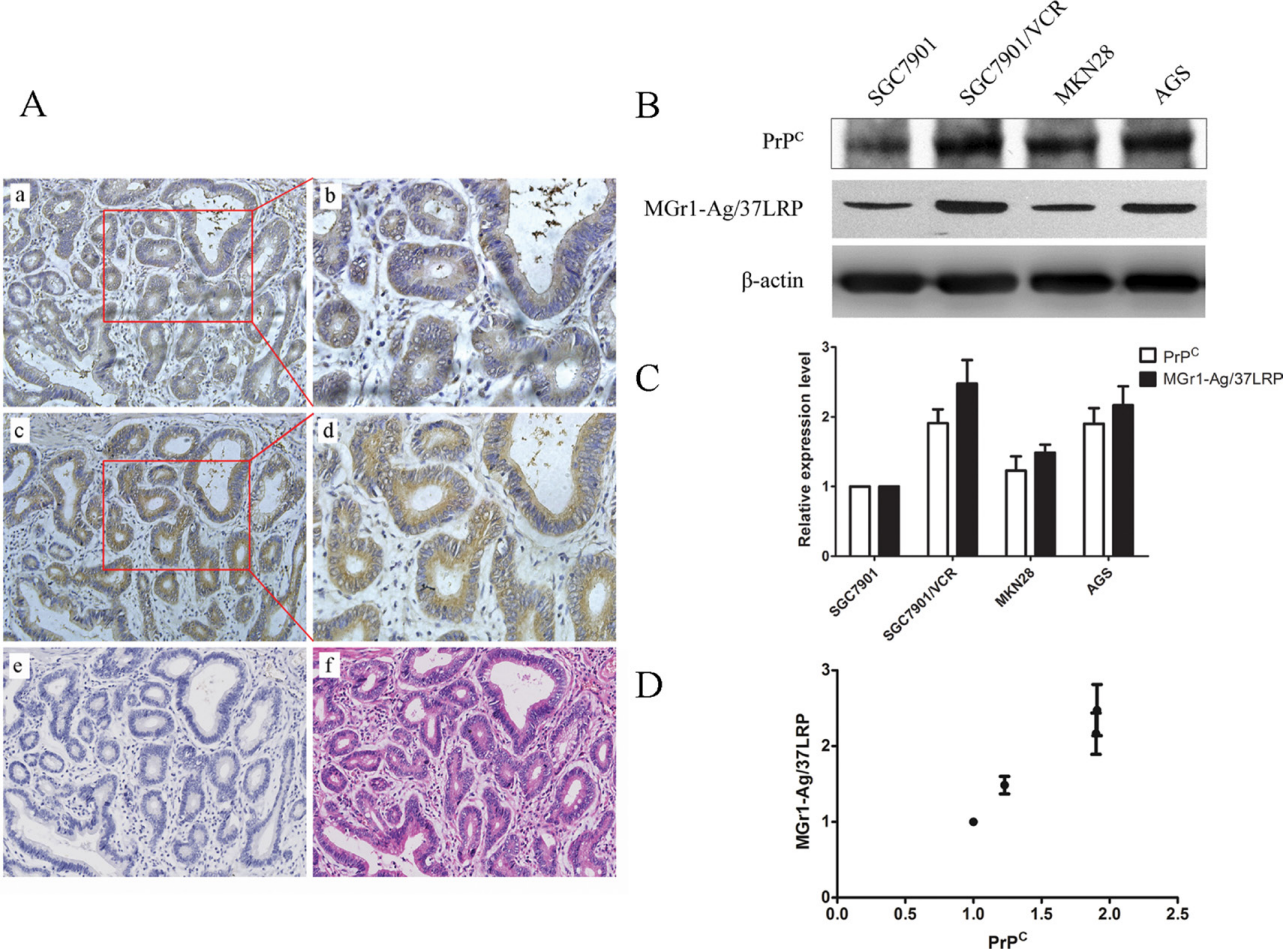
Expression pattern of PrP^C^ and MGr1-Ag/37LRP protein in gastric cancer tissue and cell lines (**A**) Results from immunohistochemical staining of MGr1-Ag/37LRP and PrP^C^ in gastric cancer tissue serial sections. Upper row: expression pattern of MGr1-Ag/37LRP in gastric cancer tissue (a 200×, b 400×); middle row: expression pattern of PrP^C^ in gastric cancer tissue (c 200×, d 400×); lower row: negative control (e 200×) and HE staining (f 200×). (**B**–**D**) Western blot analysis of PrP^C^ and MGr1-Ag/37LRP protein expression level in gastric cell lines SGC7901, SGC7901/VCR, MKN28 and AGS, β-actin was used as internal control.

Immunohistochemical staining showed a similar expression pattern of MGr1-Ag/37LRP and PrP^C^ in gastric cancer tissue serial sections (Figure 1A). MGr1-Ag/37LRP could be detected positively in 52% of the 50 patients, while PrP^C^ could be detected positively in 66% of the patients. Paired immunostaining analysis revealed a strong correlation between MGr1-Ag/37LRP and PrP^C^ expression (Table 1, *p <* 0.05). Furthermore, western blot analysis showed a significant correlative expression (*p* < 0.05) between MGr1-Ag/37LRP and PrP^C^ in gastric cancer cell lines SGC7901, SGC7901/VCR, MKN28 and AGS (Figure 1B–1D). The expression levels of PrP^C^ and MGr1-Ag/37LRP protein were both significantly higher (*p <* 0.05) in SGC7901/VCR than in SGC7901 cells (Figure 1B and 1C), indicating they may both promote the drug resistance phenotype of gastric cancer.

**Table 1:** Expression of MGr1-Ag/37LRP andPrP^C^ in gastric cancer tissue

		MGr1-Ag/37LRP (%)		Total
		+	–	
PrP^C^ (%)	+	22 (44%)	11 (22%)	33(66%)
	–	4 (8%)	13 (26%)	17(34%)
Total		26 (52%)	24(48%)	50

χ^2^ test, P < 0.05.

### Co-localization of MGr1-Ag/37LRP and PrP^C^ in gastric cancer cell lines and gastric cancer tissues

**Figure 2:**
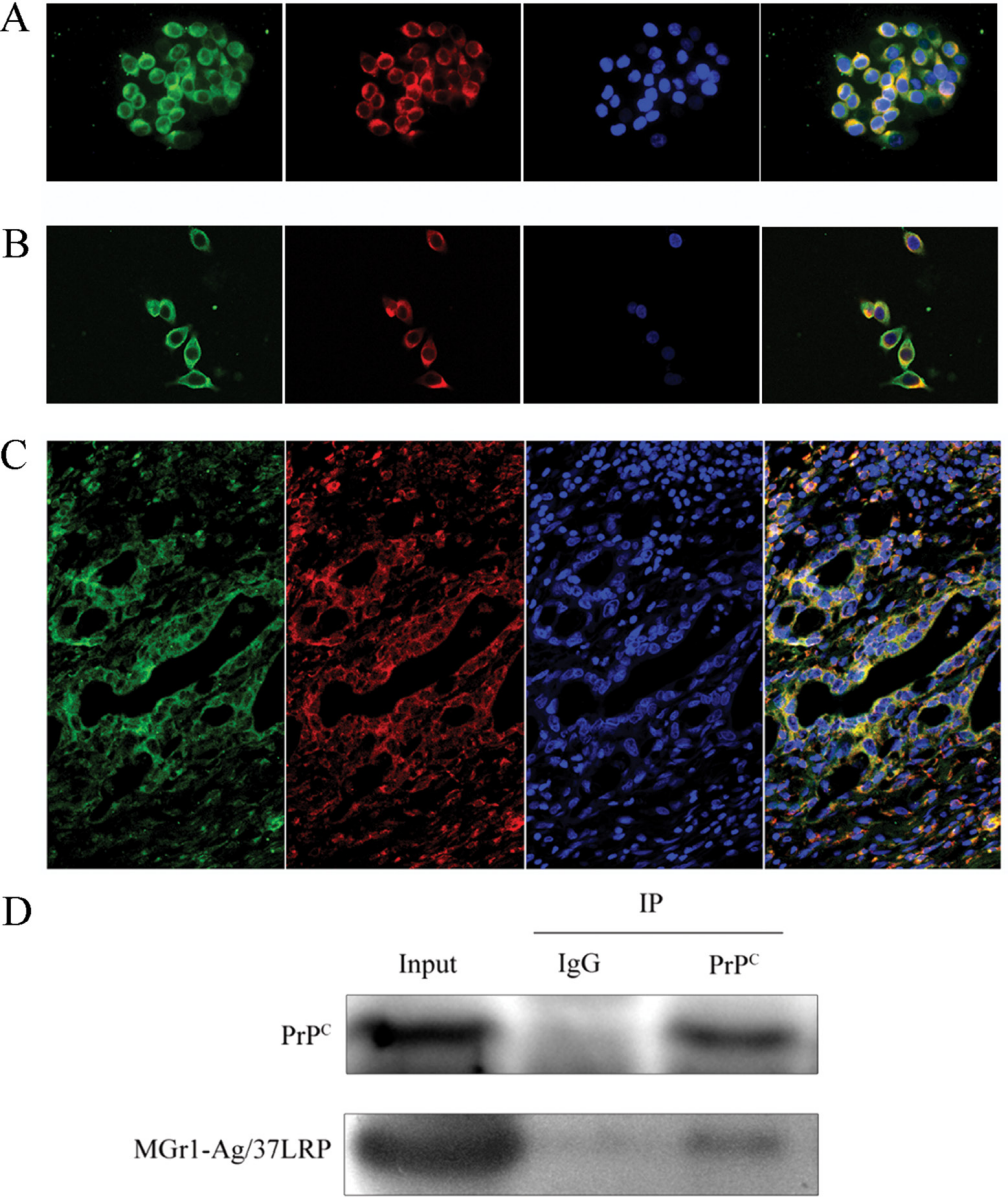
Co-localization of PrP^C^ and MGr1-Ag/37LRP in gastric cancer cell lines and tissues (**A**) and (**B**) Co-localization of PrP^C^ and MGr1-Ag/37LRP was examined by double immunofluorescence staining. Merged image (yellow) revealed co-localization between PrP^C^ (red) and MGr1-Ag/37LRP(green) in gastric cancer cell lines AGS (A) and SGC7901 (B). The nucleus was identified with DAPI (blue). (**C**) Co-localization (yellow) of PrP^C^ (red) and MGr1-Ag/37LRP (green) in gastric cancer tissues. (**D**) Interaction between PrP^C^ and MGr1-Ag/37LRP in SGC7901/VCR cells was further tested by co-immunoprecipitation using anti-PrP^C^ antibodies. Both PrP^C^ and MGr1-Ag/37LRP could be detected by their antibodies respectively in anti-PrP^C^ immunoprecipitate sample of SGC7901/VCR.

Furthermore, co-localization of PrP^C^ and MGr1-Ag/37LRP protein in gastric cancer cells and tissues was tested by immunofluorescence analysis using laser scanning confocal fluorescence assay. As shown in Figure 2A, MGr1-Ag/37LRP and PrP^C^ protein were co-localized in the cytoplasm and partially on the memberane but rare in nucleus of gastric cancer cell lines AGS and SGC7901 Figure 2A and 2B. We have noticed the abnormally high expression level of both MGr1-Ag/37LRP and PrP^C^ in the cytoplasm of gastric cancer cells, which is consistent with our previous study and results of other researchers. Castronovo V et al. reported that the majority of 37LRP was located in the cytoplasm although it had some membrane-associated domains [21]. We hypothesized such subcellular localization changes facilitates the MGr1-Ag/37LRP and PrP^C^ interaction with other intracellular proteins, which might be one of the mechanisms by which MGr1-Ag/37LRP and PrP^C^ participate in gastric cancer multi-drug-resistance. Co-localization of MGr1-Ag/37LRP and PrP^C^ protein was also observed in gastric cancer tissues glands (Figure 2C). We also confirmed the interaction between MGr1-Ag/37LRP and PrP^C^ protein by a co-immunoprecipitation assay (Figure 2D). These results provided strong evidence that these two proteins coexist in a protein complex and interact in gastric cancer cells.

### MGr1-Ag/37LRP protein expression might be regulated by PrP^C^ in gastric cancer cells

**Figure 3:**
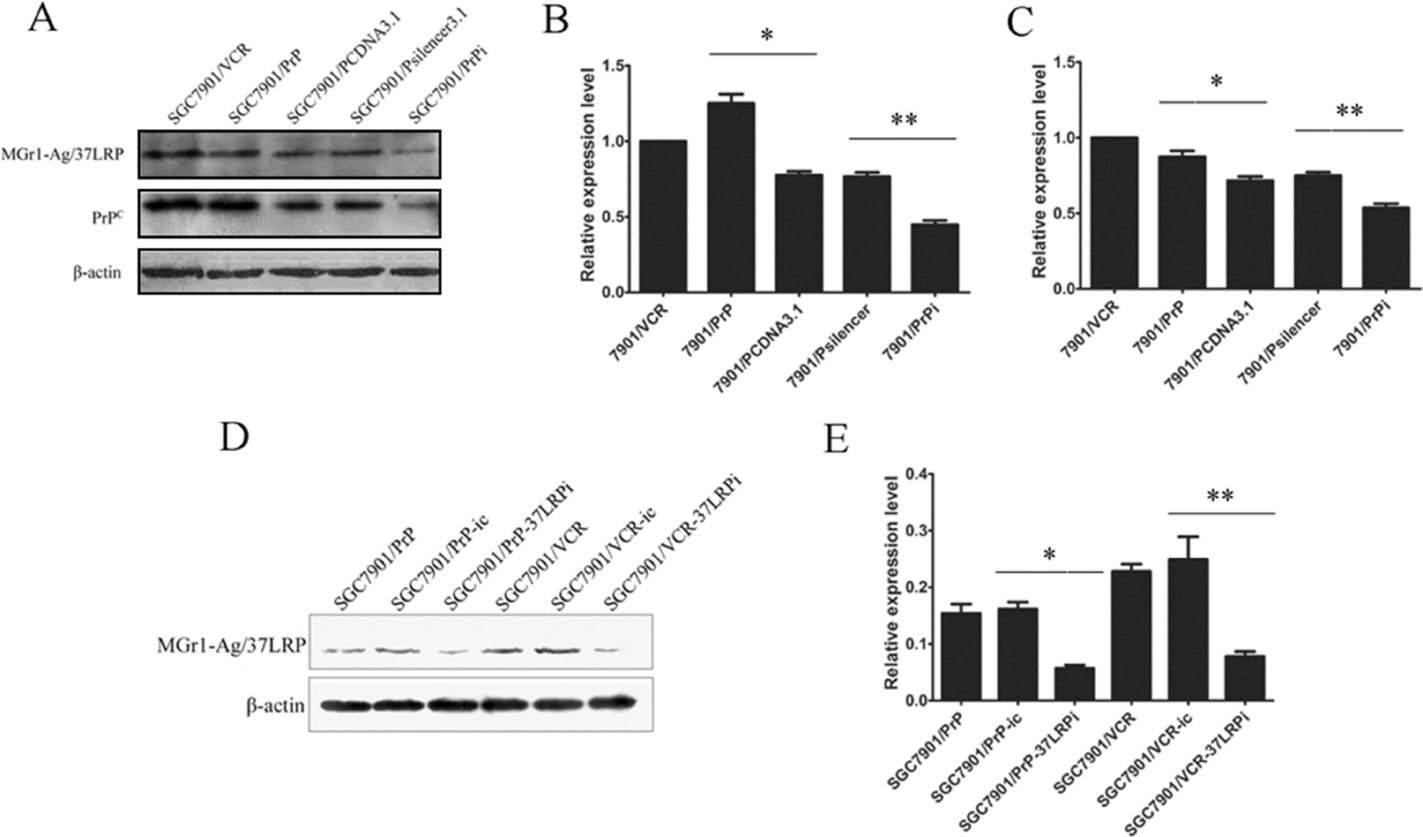
The protein expression of MGr1-Ag/37LRP in PrP^C^ up-regulated and down-regulated gastric cancer cells (**A**–**C**) Western blot analysis of MGr1-Ag/37LRP expression in PrP^C^ up-regulated and down-regulated gastric cancer cells. PrP^C^ expression in SGC7901/PrP (lane 2) increased significantly compared to SGC7901/PCDNA3.1 (lane 3) (*p < 0.05 in B) and decreased in SGC7901/PrPi (lane 4) compared to SGC7901/Psilencer3.1 (lane 5) (**p < 0.05 in B). MGr1-Ag/37LRP protein expression was also higher in SGC7901/PrP and lower in SGC7901/PrPi (A&C). SGC7901/VCR was used as positive control and β-actin as internal control. (**D**) and (**E**) Western blot analysis showed that expression level of MGr1-Ag/37LRP protein was markedly suppressed in SGC7901/PrP (lane 3) and SGC7901/VCR (lane 6) compared with siRNA negative control (lane 2 and lane 4) after transiently transfection of MGr1-Ag/37LRP siRNA (**p < 0.05 in E). β-actin was used as internal control.

We established stable transfected cell lines SGC7901/PrP and SGC7901/PrPsi in our previous work [8]. So we detected whether MGr1-Ag/37LRP protein was altered in those PrP derived gastric cancer cell lines. Western blot image (Figure 3A) and grayscale analysis (Figure 3B and 3C) showed that the expression of MGr1-Ag/37LRP protein were significantly increased in SGC7901/PrP compared with SGC7901/PCDNA3.1 and decreased in SGC7901/PrPi compared with SGC7901/Psilencer3.1 (*p <* 0.05), revealing a regulation of protein expression of MGr1-Ag/37LRP by PrP^C^ in gastric cancer cells.

**Figure 4:**
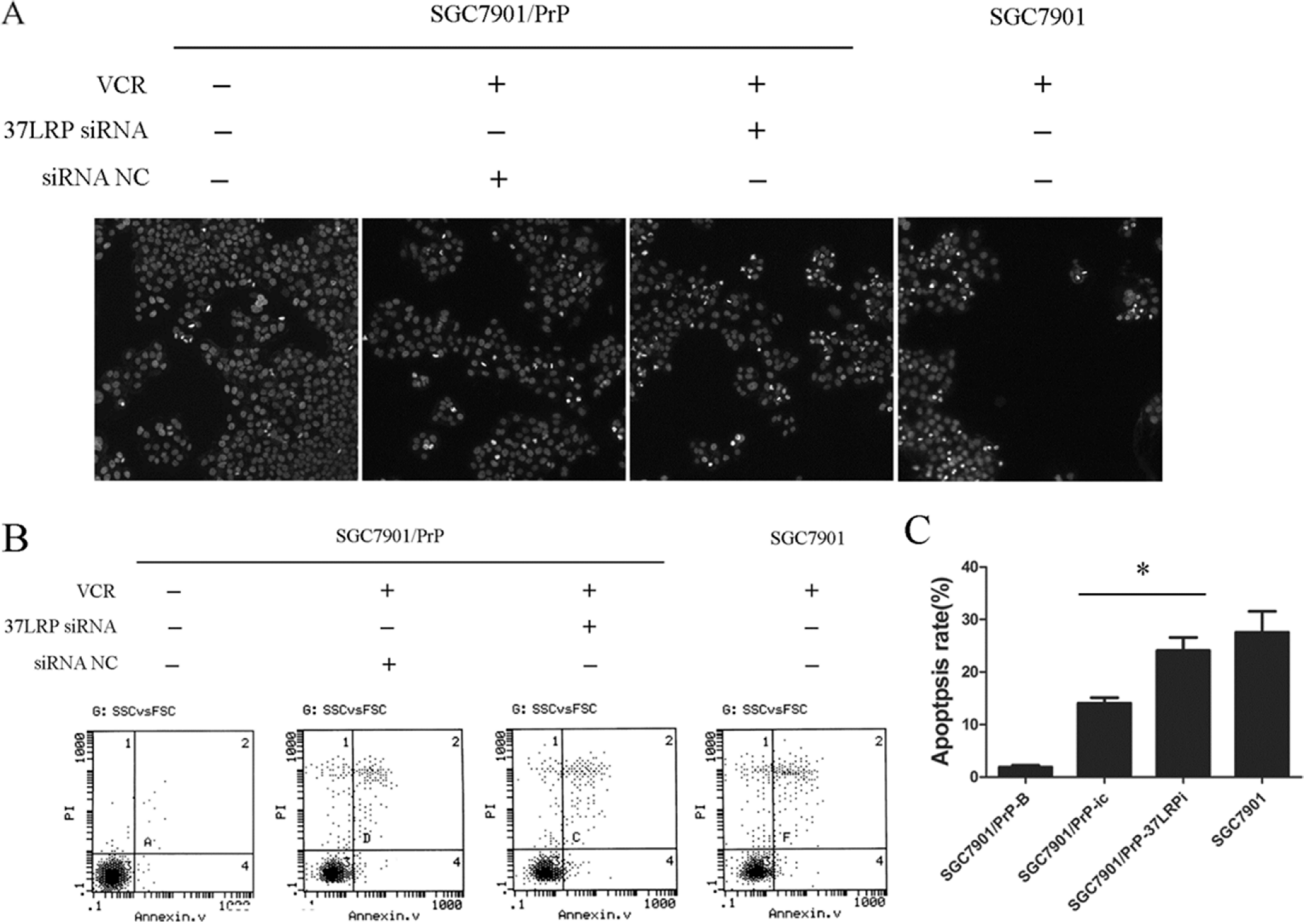
MGr1-Ag/37LRP contributes to PrP^C^ mediated gastric cancer multi-drug-resistance through promoting apoptosis (**A**) Hoechst staining results of SGC7901/PrP cells with or without MGr1-Ag/37LRP siRNA to VCR inducted apoptosis. Apoptotic cells were significantly increased in SGC7901/PrP-37LRPi compared with SGC7901/PrP-ic after treated with VCR. SGC7901 was employed as a positive control. SGC7901/PrP cells without VCR were used as a negative control. (**B** and **C**) Apoptosis rate of SGC7901/PrP cells with or without MGr1-Ag/37LRP siRNA to VCR was tested by flow cytometry. Apoptosis rate of SGC7901/PrP-37LRPi was much higher than that of control SGC7901/PrP-ic cells under the same dose of VCR treatment (*p < 0.05). SGC7901 was employed as a positive control. SGC7901/PrP cells without VCR treatment were used as a negative control. SGC7901 cells (positive control) also showed a higher apoptotic rate compared with SGC7901/PrP-ic (*p <* 0.05) but had no significant difference with SGC7901/PrP-37LRPi cells (*p >* 0.05).

### Influence of MGr1-Ag/37LRP on drug sensitivity of PrP^C^ derived gastric cancer cell lines *in vitro*

Since both MGr1-Ag/37LRP and PrP^C^ have been reported to be involved in multi-drug-resistance of gastric cancer, we explored whether MGr1-Ag/37LRP could influence the drug resistance activity of PrP^C^. SiRNA targeting MGr1-Ag/37LRP was employed as a tool for interfering with the expression of MGr1-Ag/37LRP. Western blot analysis (Figure 3D and 3E) showed that compared with cells transfected with control oligonucleotides, MGr1-Ag/37LRP protein expression was significantly decreased in the cells transiently transfected with MGr1-Ag/37LRP siRNA (*p <* 0.05). *In vitro* effects of the drugs on the growth of SGC7901/PrP-37LRPi compared with SGC7901/PrP-ic, and SGC7901/VCR-37LRPi compared with SGC7901/VCR-ic were evaluated using MTT assay respectively. As reported in Table 2, knockdown of MGr1-Ag/37LRP by siRNA could notably increase the drug sensitivity of SGC7901/PrP and SGC7901/VCR to P-gp related drugs ADR, VCR and P-gp non-related drugs 5-FU and CDDP (*p <* 0.05). SGC7901 cells were employed as a positive control and showed significantly decreased IC50 values to drugs compared to SGC7901/PrP and SGC7901/VCR (*p <* 0.05). These data suggested that MGr1-Ag/37LRP might contribute to PrP^C^-induced multi-drug-resistant phenotypes in gastric cancer cells possibly not through P-gp related pathways.

**Table 2:** MGr1-Ag/37LRP siRNA attenuates multi-drug-resistance in PrP^C^ derived gastric cancer cells (IC50 of drugs)

Cell lines	Treatment		5-FU	CDDP	ADR	VCR
	7LRP siRNA	siRNA NC				
SGC7901/PrP	–	+	0.13 ± 0.01	1.88 ± 0.26	1.57 ± 0.18	7.49 ± 1.27
	+	–	0.06 ± 0.01^†^	0.19 ± 0.06^†^	0.84 ± 0.11^†^	4.41 ± 0.19^†^
SGC7901/VCR	–	+	1.88 ± 0.57	5.27 ± 0.54	3.77 ± 0.46	17.42 ± 2.09
	+	–	0.55 ± 0.10^‡^	1.63 ± 0.28^‡^	1.94 ± 0.27^‡^	7.53 ± 0.51^‡^

^†^*p* < 0.05 vs. SGC7901/PrP cells transfected with siRNA negative control.

^‡^*p* < 0.05 vs. SGC7901/VCR cells transfected with siRNA negative control.

### MGr1-Ag/37LRP contributes to PrP^C^ mediated gastric cancer multi-drug-resistance through altering resistance ability of gastric cancer cells to drug-induced apoptosis

The suppression of drug-induced apoptosis is an important mechanism in multi-drug-resistance of cancer. We examined the effect of MGr1-Ag/37LRP on VCR-induced apoptosis in SGC7901-derived cells. Hoechst staining showed that apoptotic cells were significantly increased in SGC7901/PrP-37LRPi compared with SGC7901/PrP-ic after treatment with VCR (Figure 4A). Annexin V/PI double staining showed that apoptosis rate of SGC7901/PrP-37LRPi was much higher than that of control SGC7901/PrP-ic cells after same dose treatment of VCR (Figure 4B and 4C, p *<* 0.05). SGC7901 cells (positive control) also showed a higher apoptotic rate compared with SGC7901/PrP-ic (*p <* 0.05) but had no significant difference with SGC7901/PrP-37LRPi cells (*p >* 0.05). These results revealed that PrP contributes to a more severe resistance to VCR-induced apoptosis and synergistically functions with MGr1-Ag/37LRP in gastric cancer.

**Figure 5:**
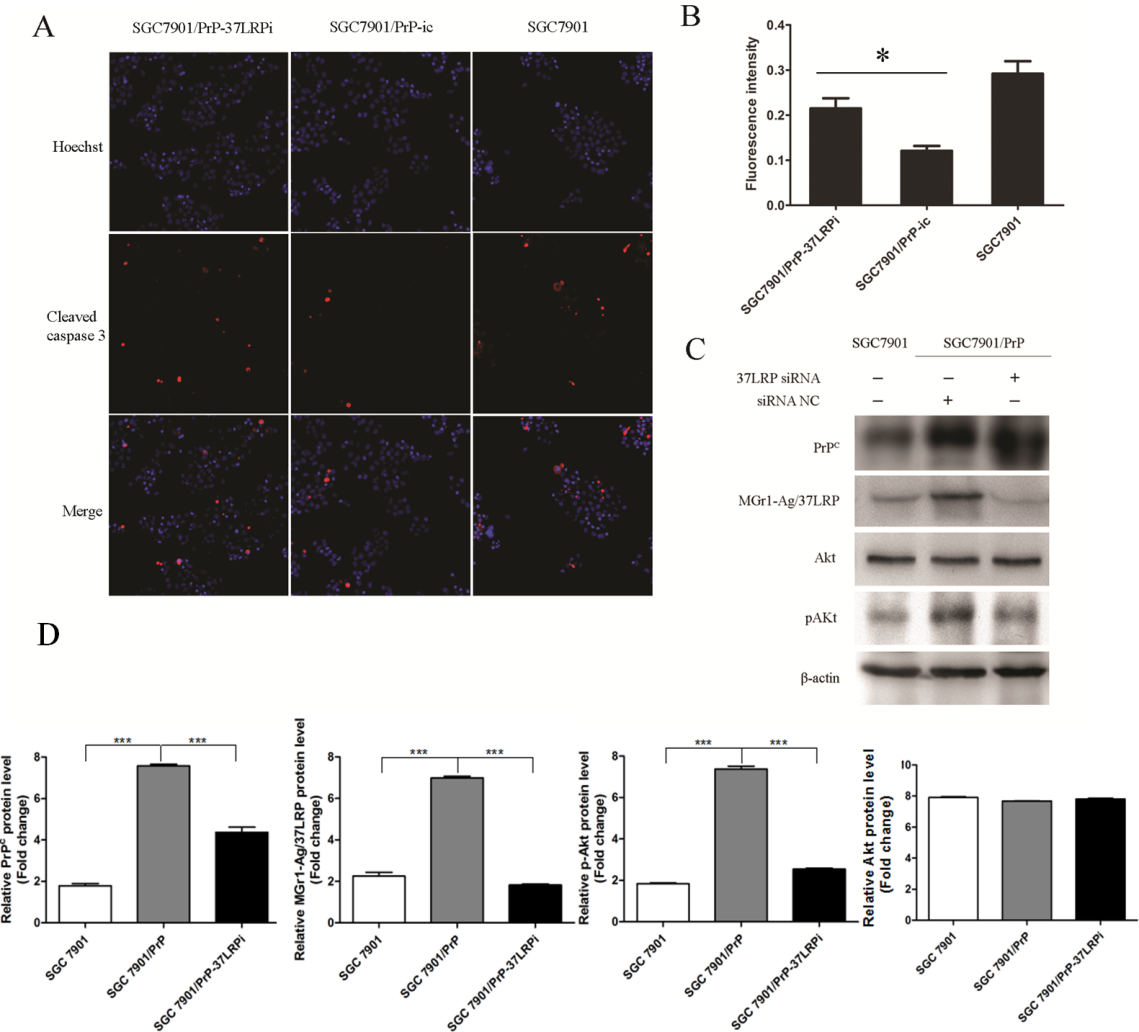
MGr1-Ag/37LRP promotes PrP^C^ induced gastric cancer multi-drug-resistance by activation of Caspase 3 probably through PI3K/AKT pathway (**A**) and (**B**) Caspase 3 activation was examined by immunofluorescence staining and the fluorescence intensity was measured using high-content screening scanning. MGr1-Ag/37LRP siRNA significantly increased caspase 3 activation in SGC7901/PrP compared with the control group (*p < 0.05). SGC7901 was employed as a positive control. (**C**) and (**D**) Western blot results showed that pAKT were significantly decreased in SGC7901/PRP when transiently transfected with MGr1-Ag/37LRP siRNA (lane 3) compared to the siRNA control group (lane 2, *p <* 0.05).

### MGr1-Ag/37LRP promotes PrP^C^ induced gastric cancer multi-drug-resistance by activation of Caspase 3 through PI3K/AKT pathway

MGr1-Ag/37LRP siRNA significantly increased caspase 3 activation in SGC7901/PrP compared with control group (Figure 5A and 5B, p *<* 0.05). PI3K/AKT signaling pathway is well-known to be critical in cell apoptosis. More importantly, PrP^C^ was verified to promote proliferation and multi-drug-resistance of gastric cancer cells though activation of PI3K/Akt pathway in our previous work [22]. So we investigated whether the PI3K/AKT pathway was regulated by MGr1-Ag/37LRP in PrP^C^ mediated gastric cancer multi-drug-resistance using western blot assay. pAKT was significantly decreased in SGC7901/PRP when transiently transfected with MGr1-Ag/37LRP siRNA compared to siRNA control group and SGC7901 cells (Figure 5C and 5D, p < 0.05). All these results suggest that MGr1-Ag/37LRP might promote PrP^C^ induced gastric cancer multi-drug-resistance by activation of Caspase 3 through the PI3K/AKT pathway.

## DISCUSSION

Gastric cancer is still one of the most common malignancies in China. Adjuvant treatments, which usually comprise chemotherapy, are the main methods for treating advanced gastric cancer patients now. But many patients die of treatment failure, which could be ascribed to a phenomenon known as multi-drug-resistance. As one of the fatal steps of tumor progression, multi-drug-resistance could be a good breakthrough point of research concerning gastric cancer treatment.

PrP^C^ and MGr1-Ag were among the 63 differentially expressed molecules isolated from drug-resistant human gastric adenocarcinoma cell line when compared to its parental cell line in our previous work [10]. Both of them were identified to be involved in promoting the multi-drug-resistance phenotypes of gastric cancer through similar signaling pathways. Overexpression of PrP^C^ in SGC7901 cells demonstrated decreased drug accumulation and increased drug release through upregulating P-glycoprotein, Bcl-2 and downregulating p53 and Bax, [12, 23, 24]. while MGr1-Ag/37LRP might also influence the multi-drug-resistance of gastric cancer cells probably by regulating the expression of P-glycoprotein, Bcl-2 and Bax [20, 25]. In our present study, the expression of these two proteins were found to correlate and co-localized in gastric cancer cell lines and gastric cancer tissues. Co-immunoprecipitation demonstrated that PrP^C^ and MGr1-Ag/37LRP interact with each other and might coexist in the protein complex. Interestingly, other researches also identified 37LRP as a ligand of the PrP^C^ in mammalian and insect cells [14, 15]. Their possible interactive site is located between aa144-179 on PrP, and between aa161-179 on 37LRP [26].

The recycle of PrP^C^ makes it a potential candidate for a ligand uptake, cell adhesion and recognition molecule or a membrane signaling molecule. In our previous work, [27] cDNA microarray analysis aiming to find PrP^C^-responsive genes in gastric cancer cells showed similar results. Fifty-two adhesion-related genes were shown to be significantly overexpressed (control signal ratio>2.0). It was reported that binding of PrP^C^ to multiple signaling molecules plays a role as a transmitter of information from the extracellular milieu to the cell that subsequently triggers the molecular cascade in multiple cell survival mechanisms. Although we still do not understand all the details, pluralism of PrP^C^ in physiological and pathological processes point to several interconnected pathways [3]. MGr1-Ag/37LRP was verified to promote PrP^C^ mediated multi-drug-resistance in this study. According to our results, siRNA targeting towards MGr1-Ag/37LRP could notably increase the drug sensitivity of PrP^C^ overexpressed SGC7901/PrP cells which showed notable drug resistance in our previous results. Further data demonstrated that MGr1-Ag/37LRP might promote the multi-drug-resistance of SGC7901/PrP mainly by enhancing anti-apoptotic abilities of gastric cancer cells through PI3K/AKT pathway. It was also reported that in cerebral ischemia, PrP^C^ deletion impairs the PI3K/Akt pathway [28]. Llorens F et. al. found that PrP^C^ could modulate epidermal growth factor receptor (EGFR) and enhances cell proliferation and cell cycle reentrance through MAPK-AKT pathway activation [29]. Similarly, in the nervous system, PrP^C^ can also mediate the activation of several signal transduction pathways, including PKA, Fyn, PI3K/Akt, and MAPK/ERK to promote neuronal survival [30, 31]. More interestingly, it was reported that IGF-1-induced enhancement of PrP^C^ expression is due to the activation of the PI3K-Akt signaling pathway.[32] Taken together, we suppose that in PrP^C^ mediated drug resistance, there may be two putative relationships between PrP^C^ and MGr1-Ag/37LRP. On the one hand, MGr1-Ag/37LRP may play a role in signal transduction in extracellular stress trans-membrane to intracellular molecular signal pathways. On the other hand, MGr1-Ag/37LRP may be a synergetic factor of PrP^C^ not only to transfer signals to downstream molecules but also acting as positive feedback to PrP^C^ through activation of PI3K/AKT pathway, thus encouraging the vicious cycle of tumorous drug resistance development. However, more evidence is needed to verify this hypothesis in future work.

As a copper-binding protein expressed predominantly in neurons, PrP^C^ could be induced in ischemic/hypoxic brain tissues and hypoxia could initiate resistance to chemotherapy by altering gene expression in solid tumors. Some transcriptional factors phosphorylated by ERK1/2 could interact with HSE in the promoter of PrP^C^ resulting in upregulation of PrP^C^ in gastric cancer cell line MKN28 during hypoxia [33]. Previous data also showed that FAK-PI3K and p42/44MAPK(ERK1/2) might be the major signaling molecules in MGr1-Ag/37LRP induced HIF-1α expression and activity which contributed to drug resistance and apoptosis resistance in gastric cancer cell lines [34]. These data supports a model of hypoxic stress reaction in gastric cancer cells, in which MGr1-Ag/37LRP might be a focal point of drug resistant signaling pathways.

There were also various studies that showed PrP^C^ as a high affinity laminin ligand for its interaction with synaptic proteins (synapsin Ib and Grb2) and cell adhesion molecules [35, 36]. There is some suggestion that PrP^C^ mediates interactions between the extracellular matrix and the neuron [37]. The 37LRP matures to a 67 kDa protein which facilitates binding of cancer cells to basement membranes [38]. We also identified a role for MGr1-Ag/37LRP in cell adhesion mediated drug resistance (unpublished data). All the data suggests an adhesion molecule related signal pathway as another candidate model of MGr1-Ag/37LRP as a focal point in promoting PrP^C^ related multi-drug-resistance in gastric cancer cells.

Since MGr1 has a novel promoting function on gastric cancer multi-drug-resistance, it could be a potential anti-tumor target which might prevent or delay drug resistance in gastric cancer. Omar et al. predicted that molecular tools such as antibodies directed against LRP/LR have the potential to act as promising alternative therapeutics for the treatment of cancer [39]. We also found that purified MGr1 antibody could significantly increase the 5-FU sensitivity of SGC7901/VCR at a concentration of 20 μg/ml (unpublished data).

Although it needs to be further confirmed *in vivo*, our findings elucidates the mechanism of how PrP^C^ triggers intracellular signaling cascade resulting in multi-drug-resistance phenotype and provides a novel candidate molecular target against gastric cancer. Combined with the earlier findings gained by our colleagues and other researchers, we propose that MGr1-Ag/37LRP may be a critical factor of several stress associated cellular signal pathways though the precise mechanisms still needs to be determined by further work.

## MATERIALS AND METHODS

### Cell culture and transfection

SGC7901/VCR cells were generated from the human gastric cancer cell line SGC7901 which was obtained from Academy of Military Medical Science (Beijing, China) by stepwise selection *in vitro*, using vincristine as inducing reagent. Both SGC7901 and SGC7901/VCR cell lines were preserved in our institute. Cells were cultivated in RPMI1640 medium (Gibco Technologies Inc.) supplemented with 10% fetal bovine serum (Hyclone), 100 U/ml penicillin, and 100 μg/ml streptomycin (Sigma-Aldrich) at 37°C with a humidified atmosphere of 5% CO_2_ and 95% air, with 1 μg/ml VCR for SGC7901/VCR.

### Clinical samples

Formalin-fixed, paraffin-embedded tumor tissue and corresponding normal tissue samples were obtained from 50 gastric cancer patients (aged between 28 to 82 years, 38 males and 12 females, 26 well differentiated and 24 moderately or poorly differentiated) who underwent surgical resection during 2005 and 2006 in Xijing Hospital, Xi'an, China. All patients had not received radiotherapy and chemotherapy before surgery. The protocols were approved by the Ethics Committee of Xijing Hospital. All cases of gastric cancer and adjacent nontumorous tissues were diagnosed clinically and pathologically.

### Immunohistochemical staining

MGr1-Ag/37LRP and PrP^C^ immunostaining were performed by an avidin-biotin method as described previously [40]. The expression level of MGr1-Ag/37LRP and PrP^C^ was determined as positive or negative by evaluating the percentage of staining tumor cells: positive for > 25% and negative for < 25%.

### Plasmid construction, siRNA and transfection

Stable transfected cell lines SGC7901/PrP and SGC7901/PrPsi were prepared in our previous work [8] by transfecting SGC7901 cells with PCDNA3.1B-PrP plasmids and Psilencer3.1-PrPsiRNA plasmids, respectively. SiRNAs of MGr1-Ag/37LRP were purchased (Shanghai GenePharma Co., Ltd) and selected using western blot (MGr1-Ag/37LRP siRNA sequence: 5′-GUGC AAUUGUUGCCAUUGA-3′-TT; negative control: 5′-UU CUCCGAACGUGUCACGU-3′-TT). Cell transfection was performed with Lipofectamine TM 2000 reagent (Invitrogen) as described in the manufacturer's protocol. Cells transiently transfected with MGr1-Ag/37LRP siRNA or negative control were defined as SGC7901/PrP-37LRPi and SGC7901/PrP-ic, respectively.

### Western blot analysis

Comparable protein samples (∼50 μg) were separated on a 12% SDS-PAGE gel and the protein-transferred nitrocellulose (Millipore) was probed with mouse anti-human antibody specific against PrP^C^ (Sigma Company, clone 3F4 bind epitopes comprising 1000), MGr1 (prepared in our lab) [18], AKT/pAKT antibodies (Cell Signaling Technology) or β-actin (Sigma Company). After three washes, the membrane was incubated with horseradish peroxidase-conjugated goat anti-mouse IgG (zhongshan golden bridge biotechnology) as secondary antibodies. The membrane was then washed again and detected using the enhanced chemiluminescence reagent (Bio-Rad Laboratories, Inc.).

### Laser scanning confocal fluorescence assay

4% paraformaldehyde-fixed SGC7901 and AGS cells plated on cover slips were permeabilized with 0.2% Triton X-100 and blocked with goat serum for 1 h at room temperature. Cover slips were then incubated with rabbit anti-human PrP^C^ antibody (1:50, Santa Cruz Biotechnology) and MGr1 antibody (1:200) at 4°C overnight. After three washes the cover slips were incubated with FITC-conjugated goat anti-mouse IgG (1:1000, Beyotime Institute of Biotechnology) and Cy3-conjugated goat anti-rabbit IgG (1:1000, Beyotime Institute of Biotechnology) as secondary antibodies for 2 h at room temperature. The nucleus were stained with DAPI. Fluorescent images were collected by the FLUOVIEW laser scanning confocal microscope (Olympus Corp).

### Co-immunoprecipitation

Approximately 5 × 10^7^ SGC7901 cells in log phase were lysated with lysis buffer (50 mmol/L Tris-Cl PH = 7.5; 150 mmol/L NaCl; 1% Triton X-100/1% NP-40; 0.5% sodium deoxycholate; 1mM Phenylmethanesulfonyl fluoride (PMSF); 1 μg/ml Aprotinin; 1 μg/ml Leupeptin) and centrifuged. Preclearing of supernatant was performed with 50 μl Protein G Plus/Protein A Agarose (Calbiochem) in dilutus buffer (50 mmol/L Tris-Cl PH = 7.5; 150 mmol/L NaCl; 0.1mg/mL PMSF; 1 μg/ml Aprotinin; 1 μg/ml Leupeptin; 0.1% NP-40). After centrifugation, the supernatant was divided into two parts. One was incubated with monoclonal mouse anti-human PrP^C^ antibody (1:50, Abcam) and the other was incubated with an independent monoclonal mouse anti-human antibody prepared in our laboratory as a negative control for 2 hours. Each of them were added to 50 μl protein A/G-agarose and incubated overnight. The precipitates were collected and carefully washed four times with the abluent buffer I (50 mmol/L Tris-Cl PH=7.5; 150mmol/L NaCl; 0.1% NP-40) and twice with the abluent buffer II (10 mmol/L Tris-Cl PH = 7.5). All the procedures above were conducted at 4°C and with gentle rotation. After centrifugation, pellets were resuspended in 40 μl SDS-PAGE sample buffer (50 mmol/L Tris-Cl pH = 8.8; 2% SDS; 0.1% Bromphenol blue; 10% Glycerol; 1% β-mercaptoethanol) and the immunocomplex supernatant was detected respectively with antibodies specific to PrP^C^ and MGr1 by western blot.

### *In vitro* drug sensitivity assay

Chemotherapeutic drugs frequently used in clinical treatment of gastric cancer (ADR, VCR, CDDP and 5-FU) were chosen to test the drug sensitivity of SGC7901/PrP and SGC7901/VCR derived transfects by the MTT assay. Cells were seeded into 96-well cell culture clusters at a density of 8 × 10^3^ cells/well and cultured for 12 hours before transfection. 12 hours after transient transfection, the medium was replaced with fresh RPMI1640 containing different drug concentrations (10×, 1×, 0.1×, 0.01×, 0×peak value of each drug in blood serum: ADR 0.4 μg/ml, VCR 0.5 μg/ml, CDDP 0.6 μg/ml, 5-FU 2 μg/ml). After 48 hours of growth in the presence of the drugs, 20 μl MTT/well reagent (5mg/ml) was added and the supernatant was discarded after further culturing for 4 hours. Adherent cells were lysed with 150 μl dimethyl sulfoxide. The absorbance of the formazan product was measured with the microplate reader (Model 680, BIO-RAD) at a wavelength of 490 nm. The relative survival rate of cell growth by different concentrations of drugs was calculated according to the following formula: (mean A490 of drug treated wells/mean A490 of untreated wells) ×100%. Finally, dose-dependent curves of drugs were drawn on semilogarithm coordinate paper and IC-50 values were determined. Each study was performed in quadruple and repeated thrice.

### Hoechest staining assay

Forty eight hours after transient transfection with MGr1-Ag/37LRP siRNA or control siRNA, respectively, SGC7901/PrP and SGC7901 cells were induced by VCR in a final concentration of 1 μg/ml for 18 hours except for blank control group. Hoechest staining was performed with a Hoechest staining assay kit (Beyotime Institute of Biotechnology) as described in the manufacturer's protocol. Each experiment was independently repeated thrice.

### Analysis of apoptosis by flow cytometer

SGC7901/PrP cells were transiently transfected with MGr1-Ag/37LRP siRNA or control siRNA, respectively, and were further cultured for 48 hours. Then SGC7901/PrP and SGC7901 cells were induced by VCR in a final concentration of 1 μg/ml for 18 hours except for blank control group. The cells were suspended in 200 μl Binding Buffer and 10 μl Annexin V-FITC and 5 μl PI were added. Apoptotic cell numbers were counted with a flow cytometer. A relative apoptotic rate was calculated according to the following formula: the amount of apoptotic cells (FITC^+^ and PI^−^) per 6000 cells/6000 cells counted randomly×100%. Each experiment was independently repeated thrice.

### Caspase 3 activation detecting and quantitating assay

Activated caspase 3 detecting and quantitating assay was performed by a high-content screening method that allows direct in-cell measurements using a fixed end-point assay based on immunofluorescence detection in cells grown on standard high-density microplates. SGC7901/PrP or SGC7901 cells were seeded into 96-well cell culture clusters at a density of 8×10^3^ cells/well and incubated for 12 hours at 37°C in 5% CO_2_. SGC7901/PrP cells were then transiently transfected with MGr1-Ag/37LRP siRNA or control siRNA, respectively, and further cultured for 48 hours. All cells were induced by VCR in a final concentration of 1 μg/ml for 18 hours. Activated caspase 3 (cleaved) detecting and quantitating assay was performed with Cellomics^®^ Caspase 3 Activation Kit (Thermo Scientific) using Thermo Scientific ArrayScan^®^ VTI HCS Reader as described in the manufacturer's protocol. Each study was performed in quadruple and independently repeated thrice.

### Statistical analysis

Numerical data are presented as the means ± SD/SEM. All statistical analyses were performed using GraphPad Prism 5. The differences between groups were assessed by the Student's *t*-test when comparing only two groups or one-way analysis of variance when more than two groups were compared. Chi-square test was used in correlation analysis of immunohistochemical results. *P <* 0.05 was considered as statistically significant and all reported *p* values were two-sided.
